# Abstract of the Proceedings of the Buffalo Medical Association

**Published:** 1856-12

**Authors:** Sanford B. Hunt


ART. II.—*Abstract of the Proceedings of the Buffalo Medical Association.*

THURSDAY EVENING, Nov. 7, 1856.

The association met.

Reading of the minutes of preceding meeting dispensed with.

Present—The President, Dr. Eastman, in the chair. Drs. Wilcox, Baker, White, Hawley, Gould, Lewis, Nichols, Miner, Newman, Dayton, Wyckoff, Lemon, Hunt, and Flint.

*Dr. Wyckoff* reported that the patient afflicted with tape-worm, whose case he had previously described to the association, had, two months since, found that more joints of the worm had passed, and had taken a other quantity of the emulsion of pumpkin seeds. At that time, he passed twelve feet of the worm. On examination, the neck of the parasite was found, but no head could be detected. Recently, he had again passed some joints, and again resorted to the emulsion of pumpkin-seeds. The result was the passage of fourteen feet of the worm, but, as before, the head was not dislodged. He has never repeated the dose of pumpkin-seed immediately after the discharge of the worm. He has probably passed in all about 100 feet of this parasite.

Dr. Wyckoff also described a case of fracture of the scapula across the angle. The subject of this accident fell a distance of about thirty-five feet, from the rigging of a vessel, and struck the deck upon the side of his head and shoulder. His head was not injured seriously, though he remained stupid some twenty-four hours before full recovery of his senses. No other injury than the fracture of the scapula was inflicted.

*Dr. Wilcox* mentioned a case of severe contusion of the back by falling upon water. A negro fell twenty-two feet from the rigging of a vessel, striking flat upon his back in the water. The concussion of the water was such as to occasion very severe contusion, and ecchymosis of the entire back.

*Prof. White* related the fall of a German hod-carrier, from the peak of a four story building to the basement, a distance of some sixty feet. On the way down, his nose struck against a beam, causing him to turn a summerset, but no injury other than that to the nose was received, and the man walked home unassisted. Also, the fall of a girl, engaged in hanging clothes upon the roof of the old American Hotel. Leaning against the balustrade, it gave way, and she sailed down to the earth upon it, unhurt. Another case was that of a young man in delirium tremens, who jumped from the fourth story of a hotel into the court, breaking only his thigh and one ankle, in the fall upon the brick pavement.

*Prof. Hunt* mentioned a patient with delirium tremens, who sprang from the third story of a building on Main street, to the pavement. A fracture of the tibia was the only immediate injury, but he was taken to the hospital and died from the combined effects of the disease and the shock. The fracture was peculiar, splitting the tibia from the ankle joint upward, for a distance of eight inches.

*Prof. White* remarked, that in contradistinction to these cases, showing how little injury might be done by falls from great heights, he could recur to numerous instances where a slip upon the pavement had broken a limb, and one in which a fractured skull, resulting in death, was occasioned by slipping on the pavement.

Other instances of similar character were mentioned, among them one of fracture of thigh from muscular effort in throwing a ten pin ball.

*Prof. Nichols* described four cases of double quotidian ague, occurring in Dr. Flint's wards at the hospital. The first case admitted did not yield readily to quinia. The patient took 10 grs. of quinine, three times daily, without benefit. Reasoning from this case, it was argued that double quotidian was more obstinate than the quotidian or tertian forms of ague; but this impression was corrected by the fact that the three other cases yielded very promptly to 5 grs. three times daily. In the cases of quotidian and tertian in the hospital, at the same time, it was found that there was a strong tendency to relapse, and that they did not yield so readily as the double quotidian. In Pennsylvania, Dr. Nichols had seen a good deal of the various forms of ague. He thought that there was a general tendency to relapse in tertian cases, more than in quotidian. The quinine given is, in many instances, impure. Very much of the cheap American quinine contains a large per centage of salicin, and is otherwise improperly manufactured. The best samples of French or English quinine could not now be bought at much below the old rates.

In answer to an inquiry, Dr. Nichols stated that he had no faith in the efficacy of cathartics before giving quinine. In Pennsylvania, where all diseases were called "bilious," he had seen almost all cases prepared for quinine by the administration of mercurial cathartics; but, so far as he had had opportunity to judge, he thought they increased the tendency to relapse.

He would mention, that in two cases of suppressed menstruation, at the hospital, paroxysms of ague had come on at the time when the menses should have made their appearance, and lasted for five or six days, passing off with the menstrual period. The ague seemed to take the place of the uterine flow.

*Dr. Wilcox* described a case of what he had at first supposed to be ague, which would not yield to quinine. The patient was pale and jaundiced, and had been four weeks in his ward, at the hospital, without improvement. He had large glandular tumors about his neck, which were uniformly worse upon the day of a chill. For the last eight or ten days he had taken a different view of the nature of the case, and had diagnosticated some form of disease at the base of the brain, in connection with the chills. He was now prescribing sol. hydrarg. biniod. cum potass. iodid., with this view. Vomiting, without assignable cause, was a symptom present, which had excited his suspicions, and he had once before seen a similar case, also accompanied by tumors on the neck, and vomitings, treated for three or four weeks as masked ague. In that case a *post-mortem* revealed large purulent deposits at the base of the brain.

And the association then adjourned, to reorganize immediately as a meeting of practitioners for business purposes.

SANFORD B. HUNT, Secretary.

